# Metformin improves cognitive impairment in patients with schizophrenia: associated with enhanced functional connectivity of dorsolateral prefrontal cortex

**DOI:** 10.1038/s41398-023-02616-x

**Published:** 2023-10-11

**Authors:** Tiannan Shao, Jing Huang, Yuxin Zhao, Weiyan Wang, Xiaohan Tian, Gangrui Hei, Dongyu Kang, Yong Gao, Fangkun Liu, Jingping Zhao, Bing Liu, Ti-Fei Yuan, Renrong Wu

**Affiliations:** 1 Department of Psychiatry, National Clinical Research Center for Mental Disorders, and National Center for Mental Disorders, The Second Xiangya Hospital of Central South University, Changsha, 410011 Hunan PR China; 2 Brainnetome Center and National Laboratory of Pattern Recognition, Institute of Automation, Chinese Academy of Sciences, Beijing, 100190 PR China; 3 School of Artificial Intelligence, University of Chinese Academy of Sciences, Beijing, 100049 PR China; 4 State Key Laboratory of Cognitive Neuroscience and Learning, Beijing Normal University, Beijing, 100875 PR China; 5 Department of Psychiatry, The First Affiliated Hospital of Zhengzhou University, Zhengzhou, 450052 PR China; 6 Department of Orthopedics, The First People’s Hospital of Changde, Changde Hospital Affiliated to Xiangya Medical College of Central South University, Changde, 415900 PR China; 7 Department of Neurosurgery, Xiangya Hospital, Central South University, Changsha, 410008 PR China; 8 Shanghai Key Laboratory of Psychotic Disorders, Brain Health Institute, National Center for Mental Disorders, Shanghai Mental Health Center, Shanghai Jiao Tong University School of Medicine, Shanghai, 200030 PR China; 9 Co-innovation Center of Neuroregeneration, Nantong University, Nantong, 226001 PR China; 10 Shanghai Key Laboratory of Anesthesiology and Brain Functional Modulation, Translational Research Institute of Brain and Brain-Like Intelligence, Shanghai Fourth People’s Hospital Affiliated to Tongji University School of Medicine, Shanghai, 200434 PR China

**Keywords:** Schizophrenia, Prognostic markers

## Abstract

Cognitive impairment is a core feature of schizophrenia, which is aggravated by antipsychotics-induced metabolic disturbance and lacks effective pharmacologic treatments in clinical practice. Our previous study demonstrated the efficiency of metformin in alleviating metabolic disturbance following antipsychotic administration. Here we report that metformin could ameliorate cognitive impairment and improve functional connectivity (FC) in prefrontal regions. This is an open-labeled, evaluator-blinded study. Clinically stable patients with schizophrenia were randomly assigned to receive antipsychotics plus metformin (*N* = 48) or antipsychotics alone (*N* = 24) for 24 weeks. The improvement in cognition was assessed by the MATRICS Consensus Cognitive Battery (MCCB). Its association with metabolic measurements, and voxel-wise whole-brain FC with dorsolateral prefrontal cortex (DLPFC) subregions as seeds were evaluated. When compared to the antipsychotics alone group, the addition of metformin resulted in significantly greater improvements in the MCCB composite score, speed of processing, working memory, verbal learning, and visual learning. A significant time × group interaction effect of increased FC between DLPFC and the anterior cingulate cortex (ACC)/middle cingulate cortex (MCC), and between DLPFC subregions were observed after metformin treatment, which was positively correlated with MCCB cognitive performance. Furthermore, the FC between left DLPFC A9/46d to right ACC/MCC significantly mediated metformin-induced speed of processing improvement; the FC between left A46 to right ACC significantly mediated metformin-induced verbal learning improvement. Collectively, these findings demonstrate that metformin can improve cognitive impairments in schizophrenia patients and is partly related to the FC changes in the DLPFC. Trial Registration: The trial was registered with ClinicalTrials.gov (NCT03271866). The full trial protocol is provided in Supplementary Material.

## Introduction

Cognitive impairment is a core schizophrenia feature and contributes remarkably to poor functional outcomes and long-term disability [1]. Alterations in multiple cognitive domains occur throughout the illness, pre-dating psychosis onset and sustaining the illness even after symptomatic remission [2]. Patients could exhibit continued, marked cognitive impairments, particularly in the domains of working memory, verbal memory, visual memory, speed of processing, and executive function. So far, no pharmacological treatment for cognitive enhancement has been approved. Therefore, developing adjunctive cognition-enhancing treatments is a high priority for the population with psychotic disorders. Improving cognitive function indicates disease regression, helps patients resume their social lives earlier, and reduces the burden on family and society [3].

Accumulating evidence suggests that metabolic disorders may accompany and act as a critical risk factor for cognitive impairment in schizophrenia [4, 5]. Metabolic syndrome (MetS) and its constituent medical criteria are prevalent in patients with schizophrenia, which is exacerbated by antipsychotic therapy [6, 7]. Our previous study reported that metformin, a well-established oral anti-diabetic drug, could effectively attenuate weight gain and metabolic disturbances in patients with schizophrenia [8, 9], and was recommended in guidelines for the management of antipsychotic-induced metabolic side effects [10]. Additionally, metformin passes through the blood-brain barrier and accumulates in brain tissues, within a few hours of administration [11]. Several studies reported the potency of metformin in improving cognition in patients with Parkinson’s disease, Alzheimer’s disease, and pediatric brain tumors [12–15]. However, its effects on cognition improvements in patients with schizophrenia are not clear. Agarwal et al. [16] evaluated the effects of metformin on improving comorbid glucose dysregulation in patients with schizophrenia spectrum disorders. Cognition was assessed as one of the secondary outcomes by the Brief Assessment of Cognition in Schizophrenia (BACS). Fourteen patients taking metformin and nine taking a placebo completed the trial, and no difference in cognition was observed between the groups. The authors noted that negative findings on cognitive parameters could be due to the small sample size. Therefore, additional clinical trials are warranted for further exploration.

The dorsolateral prefrontal cortex (DLPFC) is a critical brain region for cognitive functions [17, 18]. Substantial evidence demonstrated that DLPFC dysfunction was associated with cognitive impairments in schizophrenia, particularly when considering its connections with subcortical or cerebellar regions [19, 20]. The DLPFC serves as a central hub to recruit a distinct neural network of cognitive control [19, 21], and the deficit in DLPFC top-down modulation results in significantly reduced connectivity in patients with schizophrenia [19, 22]. The potential cognitive improvement of different domains by metformin treatment and its relation to DLPFC alterations remain to be clarified.

Here we aim to investigate if metformin administration could improve cognitive functions in patients with schizophrenia. We first conducted a randomized interventional study of 72 participants with schizophrenia to receive antipsychotics plus metformin or antipsychotics alone; we then defined relationships between metformin treatment and cognition improvement. Functional connectivity alterations of DLPFC with other brain regions were analyzed to understand the neural mechanisms related to potential changes in cognition in these subjects. The study explored whether evidence supports the use of metformin to treat cognitive deficits in patients with schizophrenia.

## Materials and methods

### Study design and participants

The study was an open-labeled, randomized, 24-week longitudinal design from September 2017 to April 2021 at the Second Xiangya Hospital of Central South University. The inclusion criteria were as follows: (1) patients with a diagnosis of schizophrenia according to the Diagnostic and Statistical Manual of Mental Disorders, Fifth Edition (DSM-5); (2) aged 18–65 who completed more than six years of primary education; (3) patients with a high risk of MetS who gained weight ≥10% of their pre-drug weight within the first year after antipsychotic medication; (4) patients with relatively stable improvement (the total score of the Positive and Negative Syndrome Scale [PANSS] ≤60); (5) disease duration ≤5 years; and (6) and received one or two stable antipsychotics medications without dosage changes during the study period.

The exclusion criteria were: (1) patients with significant neurological conditions such as Alzheimer’s disease, Parkinson’s disease, multiple sclerosis, and epilepsy was excluded from the study; (2) a history of substance abuse in the past 12 months; (3) comorbid extrapyramidal symptoms requiring additional medications; (4) received modified electroconvulsive therapy (MECT) or rTMS during the past three months, or need to start MECT/rTMS treatment during the study period; (5) comorbid physical diseases such as hepatic or renal dysfunction, diabetes, malignant tumor, or heart disease; (6) participated in a cognitive treatment program in the past three months or participated in other clinical trials; (7) pregnant or lactating.

Written, informed consent was obtained from all participants before any assessments. They then underwent a diagnostic evaluation by two psychiatrists using the Structured Clinical Interview for DSM Disorders (SCID-5) based on the DSM-5 criteria. The study was approved by the Second Xiangya Hospital Ethics Board and was conducted following the FDA-NIMH-Measurement and Treatment Research to Improve Cognition in Schizophrenia (MATRICS) Guidelines for Clinical Trial Design of Cognitive-Enhancing Drugs for Patients with Schizophrenia [23]_._

### Randomized study

Participants were randomly assigned (2:1) to receive antipsychotics plus metformin or antipsychotics alone using a computer-based random number generator. Because the treating clinicians were not blinded to the groups, we established an independent cognitive performance evaluator who was blinded to the treatment. See Supplementary Methods for sample size calculation.

Patients in the metformin group received 500 mg of metformin (Lilinghengtai, Beijing, China) three times daily as an add-on treatment for 24 weeks. The initial metformin dose was 500 mg orally in the evening for the first two days, then 1000 mg daily on day three, then 1500 mg daily on day five and thereafter. Patients who could not tolerate the maximum dose of metformin were maintained at their highest tolerated dose. The dose of metformin was chosen based on the safety and efficacy findings of a prior study of Chinese patients with chronic schizophrenia [24]. At the follow-up visits, participants were asked to return the drug package and leftover tablets. Metformin adherence was calculated as the percentage of tablets taken during the follow-up period compared to the total number of tablets that were supposed to be taken. If patients took more than 80% of their medication, they were deemed to have good adherence. Conversely, they were considered non-adherent if they took less than 80%. All patients took more than 80% of their pills, and the average medication rate for all patients was 96.1%, considered good medication adherence. The antipsychotic medications remained at a fixed dose as baseline levels throughout the course of treatment.

### Procedures

We set three time points for assessment: baseline, week 12, and week 24. Clinical information, cognitive function, blood tests, and resting-state Magnetic Resonance Imaging (MRI) were included in the assessment at each time point. Psychopathology was assessed using the PANSS and the Calgary Depression Scale for Schizophrenia. The MATRICS Consensus Cognitive Battery (MCCB), the Chinese version, was used to evaluate changes in cognitive performance and was administered by a professional evaluator (JH participated in the MCCB on-site training and passed the test organized by Prof Chuan Shi’s team at Peking University Sixth Hospital, and was responsible for the MCCB cognitive assessment for our study) [25]. The Treatment-Emergent Symptom Scale was used to monitor treatment safety to evaluate adverse events at each clinic visit.

### Metabolic-related indexes

See Supplementary Methods for more details.

### MCCB evaluation

The following seven domains were evaluated: Speed of processing (the Trail-Making Test-part A and the BACS: Symbol Coding Test, Category Fluency Test), attention and vigilance (the Continuous Performance Test-Identical Pairs), working memory (the Wechsler Memory Scale-spatial span), verbal learning (the Hopkins Verbal Learning Test-Revised), visual learning (the Brief Visuospatial Memory Test), reasoning and problem-solving (the Neuropsychological Assessment Battery-mazes), and social cognition (the Mayer-Salovey-Caruso Emotional Intelligence Test-managing emotions). After the 1–1.5-h assessment, the evaluator converted the raw scores into scale scores and then normalized the scale scores to the T-scores of related cognitive domains. The composite score is calculated as the average score of nine subtests scaled scores [25]. The seven cognitive domain T-scores of MCCB and the composite score were used in our cognition analyses. The global deficit score (GDS) with a cut-off of ≥0.5 was considered to have an overall cognitive deficit in the MCCB battery test, which is based on the average of the deficit scores corresponding to the T-scores of nine subtests [26]. Considering that there were seven participants in the antipsychotics plus metformin group and five participants in the antipsychotics alone group with GDS less than 0.5 at baseline, which indicated they might not have cognitive impairment. Hence, they were excluded from the functional MRI (fMRI) analyses and correlation analyses.

### MRI data acquisition and preprocessing

See Supplementary Methods for more detailed information on MRI data acquisition and preprocessing.

### Functional connectivity (FC) calculation and analysis

#### ROI selection

Based on our research hypothesis, we defined six ROIs of DLPFC based on a previous study based on the Brainnetome Atlas [27, 28]. The left/right DLPFC contains six ROIs, including A9/46d (area 15, area 16), A46 (area 19, area 20), and A9/46 v (area 21, area,22). All ROIs are presented in Fig. S1.

#### FC calculation

Seed reference time series of each ROI was extracted separately by averaging the time series of all voxels within the ROI. Next, Pearson’s correlation analyses were performed between the seed reference time course of each ROI and time series of the entire brain in a voxel-wise manner. To improve normality, the resultant correlation coefficients were transformed into z-values by using Fisher’s z-transformation.

**Fig. 1:**
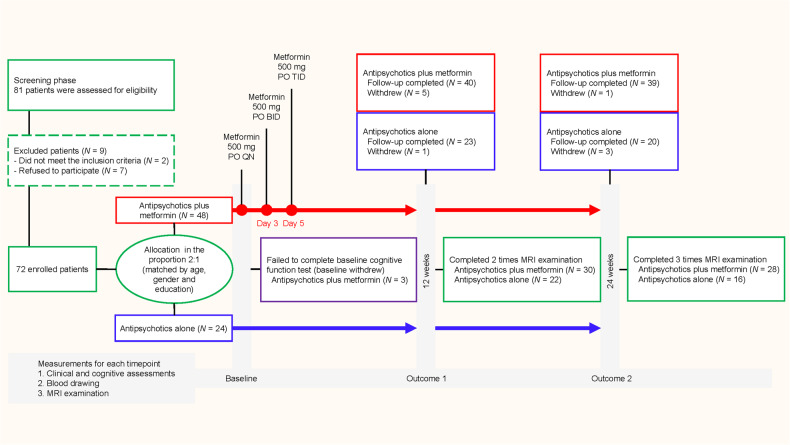
Flowchart of the trial. Eligible participants were screened and randomized to receive antipsychotics plus metformin or antipsychotics alone for 24 weeks. MRI and cognitive testing were conducted at baseline, after 12 weeks of treatment, and at the end of the trial at week 24.

For FC analysis, two-sample *t* tests were used to compare the difference of FC between groups for the six ROIs at baseline. Then we used 2 × 2 mixed model ANOVA (time × group) to compare between baseline and week 12, and between baseline and week 24, with gender, age, education and mean frame-wise displacement as covariates. In FC analysis, the cluster-level family-wise error (FWE) rates correction (cluster-wise FWE *P* < 0.05) was used for multiple comparisons with a combined individual voxel-level threshold of uncorrected *P* < 0.001. Subsequently, FC values would be extracted from the significant regions derived from the time × group interaction effects for further analysis. Due to COVID-19 epidemic restrictions in China, eight patients were not able to complete the entire MRI examination at week 24 (Fig. 1).

We then performed mediation analyses to evaluate the relationship among metformin treatment, MCCB scores, and FC changes using BruceR packages (R version 4.2.0, https://psychbruce.github.io/bruceR/). The mediation model included treatment group (binary variable) as the predictor (x), MCCB scores at follow-up as the outcome variable (y), and FC changes from week 12 to baseline (Δ_12-0_) as the mediator (m). Age, gender, years of education were included in the analysis as covariates [29].

### Statistical analysis

Statistical analyses were performed using SPSS version 22.0. Continuous variables are presented as mean with standard deviation (SD). The results of the test of homogeneity of the variances between groups in the Supplementary Results. Two-sample *t* tests, chi-square tests, and Mann–Whitney *U* tests were used for baseline intergroup comparisons. Dose equivalents of antipsychotics were used chlorpromazine (CPZ) equivalents based on defined daily doses (DDD) method [30]. We used a linear mixed-effects model to assess the metformin effect to accommodate missing values from the follow-up period. The unstructured covariance structure was selected to model the residual covariance matrix. Additionally, group and time points were considered fixed factors, and the baseline variables (gender, age, education, duration of illness, antipsychotics dose equivalents (CPZ-DDD), and the relevant baseline outcome scores) were regarded as covariates. We also analyzed the time × group interaction effects. The simple effect of time and the simple effect of group were also evaluated using the least-significant-difference method respectively. An intention-to-treat approach was applied for comparisons of metabolic measurements, paired *t* test or Wilcoxon signed-rank test was used to analyze the differences between baseline and two-time points within the group, and analysis of variance was used to examine the differences in metabolism measurements between two groups over a 12-week period and over a 24-week period. Pearson correlation analyses were used to analyze the correlations of change in MCCB cognitive scores, changes in MRI measurements, and changes in metabolism parameters. The chi-square test was used to compare adverse events between groups. A two-sided significance level was set at *P* < 0.05.

## Results

### Description of baseline demographic, clinical, and cognitive data

As shown in Fig. 1, 72 patients were randomly assigned to the two treatment groups. 59 patients (82%) completed the 24-week treatment. The mean age was 22.8 (SD = 4.8), and the mean treatment duration was 24.0 months (SD = 15.7). The mean PANSS total score was 42.48 (SD = 5.2), and the mean MCCB composite score was 39.4 (SD = 6.1). The antipsychotics plus metformin group and the antipsychotics alone group were balanced regarding demographics, antipsychotic medication, symptom scales, and cognitive performances at baseline (Table S1).

### Metformin ameliorated cognitive dysfunction as evaluated by the MCCB in patients with schizophrenia

**Fig. 2:**
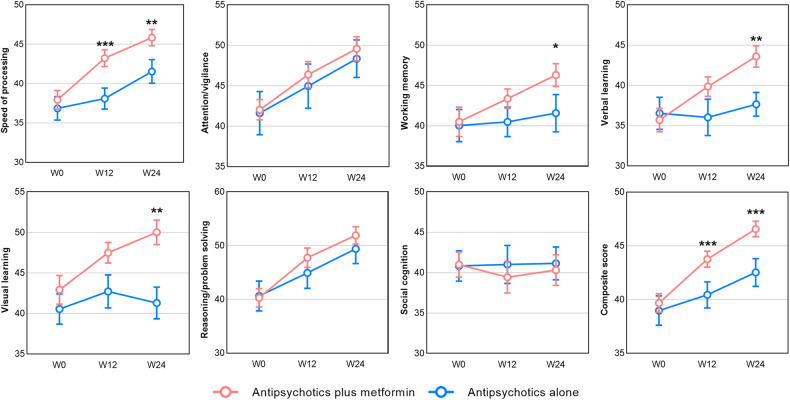
Cognitive performance of MCCB at each time point between the antipsychotics plus metformin group and the antipsychotics alone group. Values are presented as mean and standard error estimated from linear mixed-effects models. Abbreviations: MCCB, MATRICS Consensus Cognitive Battery; W0, baseline; W12, week 12; W24, week 24. For the simple effect of metformin treatment at week 12/24 based on linear mixed-effects models, *P < 0.05, **P < 0.01, ***P < 0.001.

MCCB cognitive scores of each time point in the two groups are shown in Fig. 2. After 24-week treatment, greater improvements were observed in the MCCB composite scores in the antipsychotics plus metformin group (treatment group) than in the antipsychotics alone group (control group) (estimated mean difference = 3.43, 95% CI: 1.83 to 5.02; *P* < 0.001). Significant improvements in the following domains were observed in the metformin group compared with the control group: speed of processing (estimated mean difference = 3.75, *P* = 0.002), working memory (estimated mean difference = 4.39, *P* = 0.020), verbal learning (estimated mean difference = 5.71, *P* = 0.003), and visual learning (estimated mean difference = 7.08, *P* = 0.004; Table S2). *Post-hoc* responder analysis showed that after 24 weeks of treatment, metformin significantly increased the likelihood of a ≥ 5-point improvement in the MCCB composite score versus control (OR: 5.94, 95% CI: 1.82 to 19.40), which was considered as significantly improved cognitive performance. More patients in the metformin group had a ≥ 5-point improvement in this score than in the control group (28/39, 71.8% versus 6/20, 30%, respectively), which is a significant difference (χ^2^ = 9.46, *P* = 0.002). In the secondary analysis, working memory, verbal learning, and visual learning were significantly improved in the treatment group after 24 weeks but did not change in the control group (Table S3).

We further analyzed the improvement of metformin on cognitive function in the participants with GDS ≥ 0.5, which demonstrated overall cognitive impairment. The treatment group exhibited greater improvement in MCCB composite score than the control group (estimated mean difference = 4.05, 95% CI: 2.33 to 5.77, *P* < 0.001). The treatment group also presented a significant increase in speed of processing (estimated mean difference = 4.60, *P* = 0.001), working memory (estimated mean difference = 4.11, *P* = 0.040), verbal learning (estimated mean difference = 6.94, *P* < 0.001), and visual learning (estimated mean difference = 7.95, *P* = 0.002) than the control group after 24-week metformin treatment, which was consistent with the results of the previous analyses including all participants (Table S4–S5).

### Metformin improves metabolic disturbance which is correlated with cognitive improvement

At week 12, those in the antipsychotics plus metformin group had significant decreases in weight, BMI, total cholesterol, LDL-C, fasting glucose, insulin, and IRI levels. However, all these metabolic outcomes did not change in the antipsychotics alone group. Significant differences were observed in the changes in weight, BMI, and IRI levels between the two groups over 24 weeks (Table 1).

**Table 1:** Metabolic measurements of two groups at each time point and comparisons of changes over 12 weeks and over 24 weeks of all metabolic outcomes based on ITT method.

	Antipsychotics plus metformin (*N* = 40) Mean (SD)	Antipsychotics alone (*N* = 23) Mean (SD)	*F*^a^	*P*^a^
Weight, kg				
Baseline	67.45 (11.49)	65.27 (9.30)	-	-
Week 12	66.00 (11.17)**	65.31 (9.80)	4.035	0.049
Week 24	65.58 (11.17)**	65.77 (9.67)	4.945	0.030
BMI, kg/m^2^				
Baseline	25.83 (3.89)	24.39 (2.86)	-	-
Week 12	25.29 (3.85)**	24.36 (2.76)	3.432	0.069
Week 24	25.12 (3.75)**	24.51 (2.44)	4.095	0.047
Triglyceride, mmol/L				
Baseline	1.47 (0.77)	1.48 (0.76)	-	-
Week 12	1.55 (1.00)	1.34 (0.93)	0.978	0.327
Week 24	1.45 (0.70)	1.91 (2.86)	0.994	0.323
Total cholesterol, mmol/L				
Baseline	4.54 (0.78)	4.24 (0.61)	-	-
Week 12	4.21 (0.74)***	4.31 (0.58)	8.128	0.006
Week 24	4.26 (0.74)**	4.18 (0.74)	1.995	0.163
HDL-C, mmol/L				
Baseline	1.28 (0.22)	1.20 (0.28)	-	-
Week 12	1.20 (0.22)***	1.20 (0.28)	5.388	0.024
Week 24	1.28 (0.57)*	1.16 (0.27)	0.082	0.776
LDL-C, mmol/L				
Baseline	2.74 (0.66)	2.65 (0.49)	-	-
Week 12	2.52 (0.67)**	2.67 (0.41)	3.749	0.057
Week 24	2.58 (0.66)*	2.55 (0.56)	0.191	0.664
Fasting glucose, mmol/L				
Baseline	4.97 (0.36)	4.81 (0.58)	-	-
Week 12	4.83 (0.43)*	4.75 (0.62)	0.494	0.485
Week 24	4.78 (0.48)*	4.71 (0.47)	0.664	0.418
Insulin, mU/L				
Baseline	17.74 (8.32)	14.54 (6.94)		-
Week 12	15.98 (6.95)*	17.87 (14.78)	2.369^b^	0.137
Week 24	16.02 (7.17)	20.17 (15.28)	4.108^b^	0.053
IRI				
Baseline	4.11 (1.93)	3.14 (1.77)	-	-
Week 12	3.47 (1.52)***	3.78 (2.91)	3.124^b^	0.089
Week 24	3.51 (1.65)*	4.15 (2.95)	4.764^b^	0.038

For *P*-values from comparisons between each time point and baseline within each group that were obtained by the paired t test or Wilcoxon signed-rank test, * *P* < 0.05, **P < 0.01, ***P < 0.001.

*ITT* intent-to-treat analysis, *BMI* body mass index, *HDL-C* high-density lipoprotein cholesterol, *LDL-C* low-density lipoprotein cholesterol *IRI* insulin resistance index, *SD* standard deviation, *CI* confidence interval.

^a^*F*-values and *P*-values were derived using ANOVA to compare the difference between the means of the two groups from each time point to baseline.

^b^The Welch ANOVA was used to compare the values with unequal variances between the two groups.

**Fig. 3:**
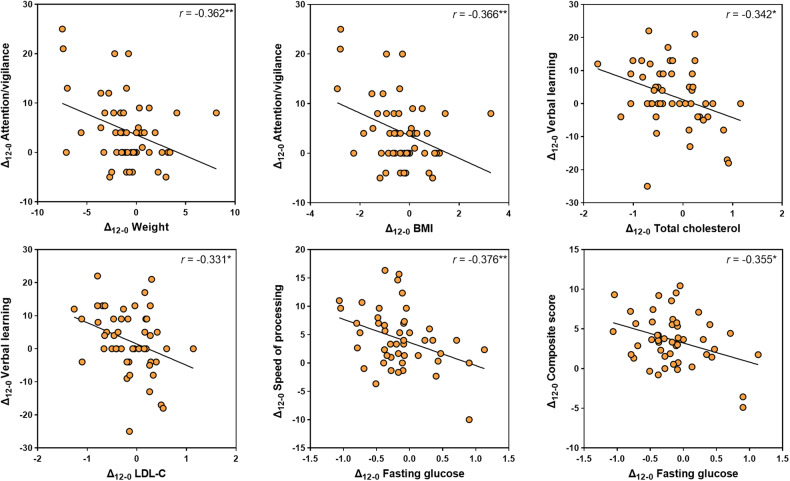
Correlations between changes in metabolic measurements and changes of MCCB cognitive scores from baseline to week 12. Δ_12-0_ indicates changes from baseline to week 12. Abbreviations: MCCB, MATRICS Consensus Cognitive Battery; BMI, Body Mass Index; LDL-C, low-density lipoprotein cholesterol. *P < 0.05, **P < 0.01.

At week 12, the increases in weight and BMI were significantly negatively correlated with the improvement of attention/vigilance (weight, *r* = −0.362, *P* = 0.009; BMI, *r* = −0.366, *P* = 0.008). The elevation in fasting glucose was negatively correlated with the improvement of speed of processing (*r* = −0.376, *P* = 0.009) and MCCB composite score (*r* = −0.355, *P* = 0.013). The increases in LDL-C (*r* = −0.331, *P* = 0.019) and cholesterol (*r* = −0.342, *P* = 0.014) was negatively correlated with verbal learning (Fig. 3). The correlations between attention/vigilance and weight/BMI were still significant over 24 weeks (weight: *r* = −0.298, *P* = 0.040; BMI: *r* = −0.302, *P* = 0.037).

### The alteration of the DLPFC after metformin treatment and its relationship with cognition improvements

**Fig. 4:**
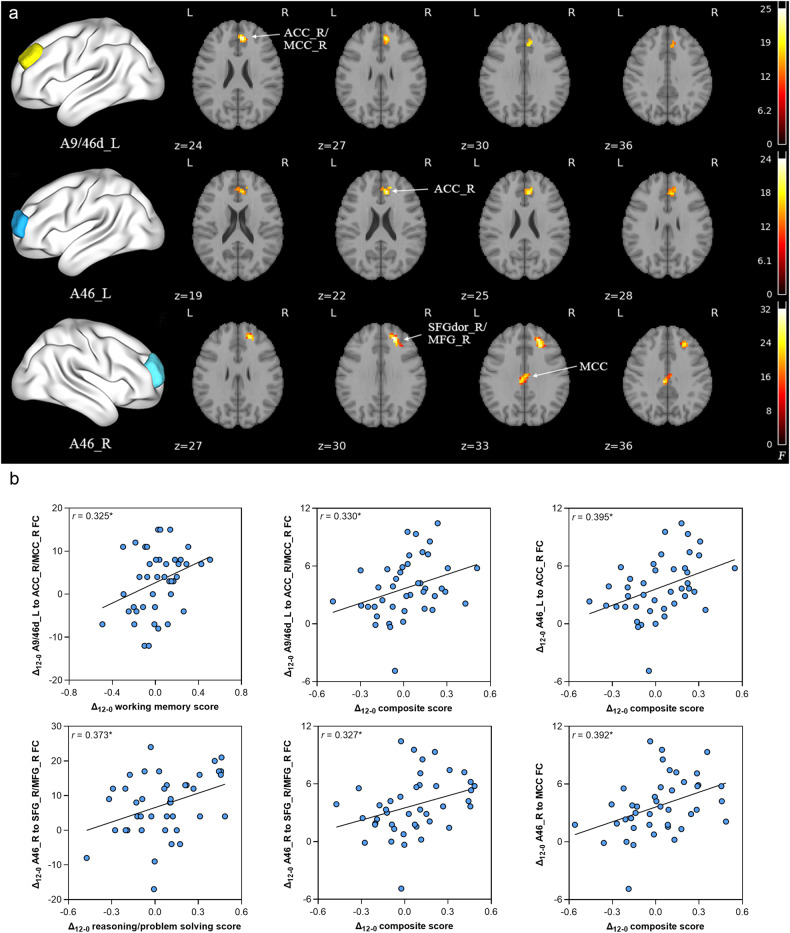
Longitudinal changes of ROI-based voxel-wise FC in the antipsychotics plus metformin group compared with the antipsychotics alone group over 12 weeks and correlations with MCCB composite score. **a** Significant clusters showing time × group interaction effects in FC with DLPFC subregions as seeds between two groups from baseline to week 12 (cluster-wise FWE *P* < 0.05). **b** Correlations between changes of FC and MCCB composite score. Δ_12-0_ indicates changes of measurements between week 12 and baseline. Abbreviations: FC functional connectivity, MCCB MATRICS Consensus Cognitive Battery, DLPFC dorsolateral prefrontal cortex, FWE family-wise error, ACC anterior cingulate cortex, MCC middle cingulate cortex, SFG superior frontal gyrus, MFG middle frontal gyrus, L/R left/right. *P < 0.05.

After 12 weeks of treatment, we found significant time × group interaction effects on voxel-wise FC with the DLPFC subregions as seeds between the metformin group and the control group. These FC include as follows: left A9/46d to right anterior cingulate cortex/middle cingulate cortex (ACC/MCC); left A46 to right ACC; right A46 to right superior frontal gyrus/middle frontal gyrus (SFG/MFG), and to MCC (Fig. 4a, Table S6–S7).

The FC with left DLPFC A9/46d to right ACC/MCC, left A46 to right ACC, right A46 to right SFG/MFG and MCC were significantly correlated with MCCB composite score and several domains (Fig. 4b, Table S8).

**Fig. 5:**
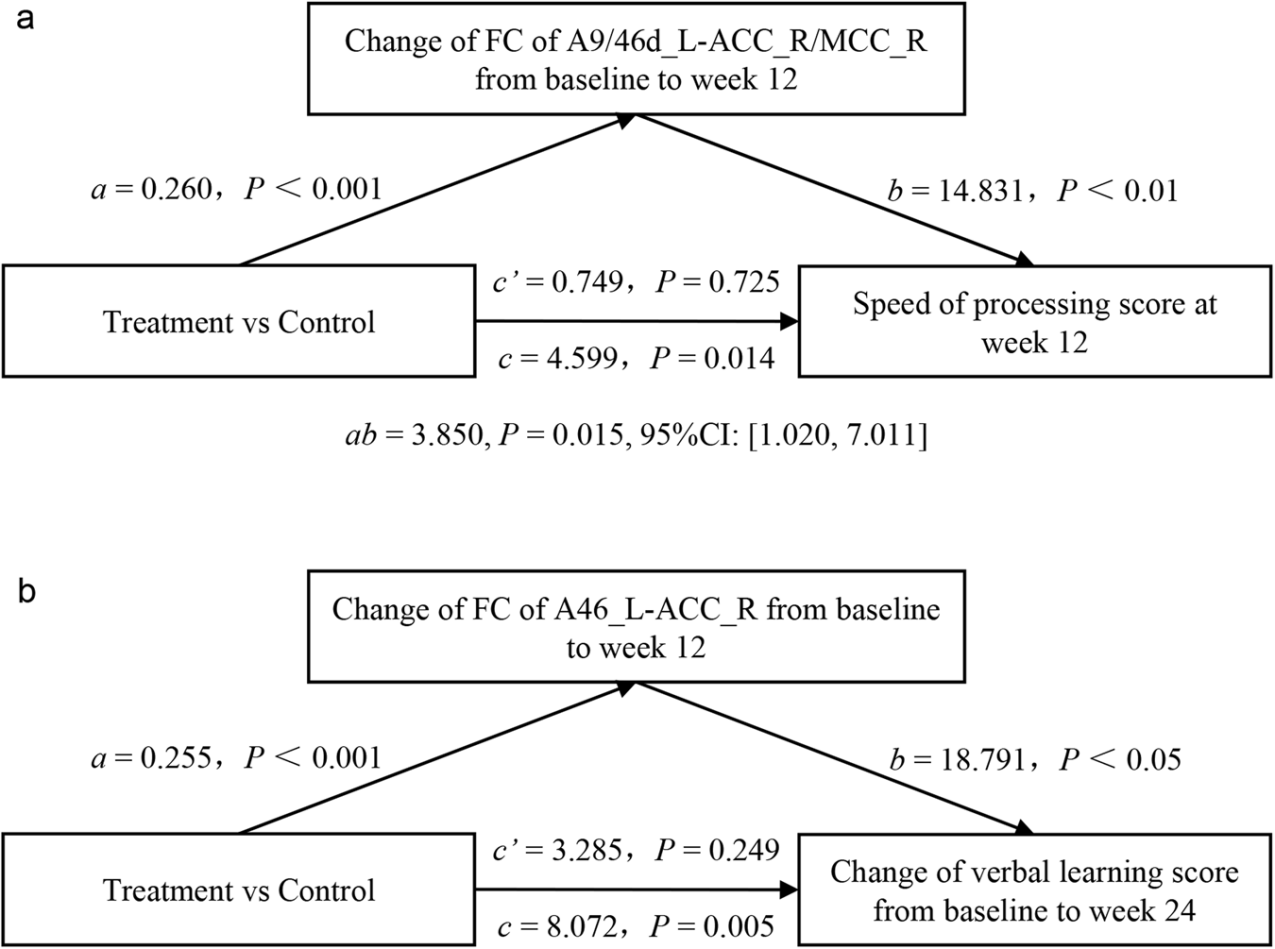
Mediation analysis showing how FC changes between the DLPFC and other brain regions mediate the effect of metformin on cognitive improvement. **a** A significant positive direct effect of metformin treatment on speed of processing improvement in the follow-up visits as well as indirect effect via the FC between left DLPFC A9/46d to right ACC/MCC and **b** a significant positive direct effect of metformin treatment on verbal learning improvement in the follow-up visits as well as indirect effect via the FC between left A46 to right ACC. The figure shows path coefficients and corresponding *P* values; path *a* indicates the effect of metformin treatment on the FC changes between the DLPFC and other brain regions after treatment; path *b* indicates the effects of FC changes on cognitive changes; path *c’* indicates the direct effect of metformin treatment on cognitive improvement; Indirect effect coefficient *ab* indicates the mediating effect. Abbreviations: FC, functional connectivity; DLPFC, dorsolateral prefrontal cortex; ACC, anterior cingulate cortex; MCC, middle cingulate cortex; L/R, left/right.

Mediation analyses were performed to test whether the FC changes between DLPFC and other regions served as a mediator between metformin treatment (independent variable) and MCCB improvements (dependent variable). This analysis revealed a significant positive direct effect of metformin treatment on speed of processing improvement in the follow-up visits as well as indirect effect via the FC between left DLPFC A9/46d to right ACC/MCC (Fig. 5a); a significant positive direct effect of metformin treatment on verbal learning improvement in the follow-up visits as well as indirect effect via the FC between left A46 to right ACC (Fig. 5b).

### Safety evaluation

After 24 weeks, the most frequently observed adverse event was decreased appetite (15/45, 33.3% in the antipsychotics plus metformin group vs. 0% in the antipsychotics group, χ^2^ = 10.222, *P* = 0.001). No significant intergroup differences were observed in other adverse events (Table S9). More details were shown in Supplementary Results.

## Discussion

To our knowledge, this is the first clinical proof that metformin could significantly improve cognitive impairments in patients with schizophrenia, especially in speed of processing, working memory, verbal learning, and visual learning. Few studies investigating pharmacological treatments for cognitive impairment in schizophrenia report positive findings [1, 31]. Twelve-week treatments with the glycine transporter inhibitor BI 425809, the 5-HT2A and sigma-2 receptor antagonist roluperidone (MIN-101), and the α7 nicotinic acetylcholine receptor agonist encenicline significantly improved cognition in three large double-blind phase-2 studies. The efficacy of metformin therapy increased with duration; the mean change of MCCB composite score had continued significant improvements during the two follow-up time points. However, in previous studies, the baseline MCCB composite score was less than that of our study, which may be due to the greater average participant age and treatment durations in those studies. Among the seven cognitive sub-domains of the MCCB, the largest intergroup differences were observed in visual learning, verbal learning, working memory, and speed of processing. The improvement in working memory due to metformin that we observed is consistent with previous observations that metformin demonstrated better performance than placebo on working memory in survivors of pediatric brain tumors [12, 32]. To avoid the potential influence of ceiling effects, we analyzed patients with a GDS cut-off ≥0.5, which was used for the classification of overall impairment status [33]. The results in this group were somewhat stronger for cognitive effects of metformin but generally consistent with the earlier analysis of the entire sample.

Nevertheless, not all metformin studies have yielded significant improvements. Despite the previously reported correlations between metabolic dysfunction and cognition [34], Hartman et al. [35] did not observe significant cognitive improvements in overweight postmenopausal breast cancer survivors after metformin treatment. Metformin was useful to ameliorate metabolic dysfunction, the improvement of cognition performance was correlated with the controlling of metabolic dysfunction in our study. However, only increases in weight and BMI negatively correlated with the improvement of speed of processing and attention/vigilance at week 24. In schizophrenia, the long-term use of antipsychotics greatly increased the risks of appetite gain and metabolic dysfunctions, which may be related to worse cognitive performance [36]. The precise mechanism underlying how metformin can improve cognitive dysfunction in patients with schizophrenia remains complex and unclear. The complex pattern of cognition implies that metabolic state should be considered in attempts to elucidate the biological mechanisms of impaired cognition in schizophrenia. As cognitive performance evaluation could be complicated by symptom fluctuations [23], we also evaluated the changes in psychiatric symptoms by PANSS, and no significant group differences were observed. Therefore, the efficacy of metformin in cognitive impairment is separate from its effect on clinical improvement.

Another important finding of our study was that metformin enhanced the FC between the DLPFC subregion and the anterior and middle cingulate cortex (ACC/MCC) as well as enhancing the FC between the DLPFC subregions. Furthermore, the correlation analyses and mediation analyses indicated that the efficacy of metformin for cognitive impairment in patients with schizophrenia might be achieved by enhancing the FC between DLPFC and ACC/MCC. The DLPFC is a key node of the frontoparietal network [37], it is involved in a variety of cognitive processes such as attention, decision-making, working memory, and emotion regulation [38]. Task-related fMRI study has found that patients with schizophrenia showed reduced activation of DLPFC during an episodic memory task [39]. In addition, resting-state fMRI showed that reduced FC between DLPFC and caudate in patients with schizophrenia was associated with a deficit of executive function [40]. The ACC, which belongs to the salience network [41], receives afferent information from multiple brain regions, is involved in reward, motivation, decision-making, learning, executive function, and emotion-related cognitive processes [42]. Neuroimaging changes such as reduced gray matter volume and FC abnormalities have been reported in ACC of patients with schizophrenia [43, 44]. During the variable attention and congruency task, patients with schizophrenia exhibited abnormal functional coupling between DLPFC and ACC compared with healthy controls [45]. Moreover, previous studies reported that reduced fractional amplitude of low-frequency fluctuations in ACC was associated with poorer working memory in patients with schizophrenia [46]. Moreover, MCC plays a vital role in attentional control, response selection, regulation of the autonomic nervous system, reward behavior, and spatial perception [47, 48]. Combined with the above evidence, it is reasonable to suggest that the enhancement of functional coupling between DLPFC and ACC/MCC by metformin may be a potential neurobiological mechanism for the improvement of cognitive function in patients with schizophrenia. Further study is warranted to explore the comprehensive mechanism of metformin for improving cognition dysfunction by combining multimodal MRI and molecular biological techniques.

This study has some limitations. First, it was not a double-blind study, which may have caused bias and overestimated the effect of metformin on improving cognitive function. Our preliminary evidence of efficacy requires confirmation in a multicenter, double-blind, placebo-controlled trial. Second, we confined the analysis to the DLPFC and did not examine other brain areas known to be recruited into a network for cognitive performance. Third, although the effects of metformin on cognition and on metabolism are correlated, subsequent studies are needed to demonstrate a possible causal relationship. At last, we did not administer daily functioning or quality-of-life scales, which may have led to a conceptually narrow evaluation of cognition.

The present study found that metformin effectively improves cognition in patients with schizophrenia. The fMRI activity index in the DLPFC increases with metformin treatment and correlates with cognitive performance. More than 50% patients with schizophrenia had metabolic dysfunction, including weight gain, hyperglycemia, dyslipidemia, and insulin resistance [49]. Due to the high prevalence and serious health consequences of metabolic disturbances, metformin can be a promising choice due to its dual capacity as a cognitive enhancer and metabolic corrector. During the trial, metformin treatment was safe and few side effects were reported. Although we conclude that larger confirmatory clinical trials are warranted, the present study clearly suggests an alternative pharmacological treatment for cognitive impairment, notably in patients with comorbid metabolic disturbances.

### Supplementary information

Academic Journals Reporting Checklist

Supplemental Meterials

## Data Availability

The data that support the findings of this study are available on request from the corresponding author.
