# Supplementary material for: Metformin improves cognitive impairment in patients with schizophrenia: associated with enhanced functional connectivity of dorsolateral prefrontal cortex
Source: Transl Psychiatry. 2023 Oct 11;13:315. doi: 10.1038/s41398-023-02616-x (PMC10567690; doi:10.1038/s41398-023-02616-x)
Supplement: Supplementary file 2 — Supplemental Meterials [file 41398_2023_2616_MOESM2_ESM.docx]

**Metformin improves cognitive impairment in patients with schizophrenia: associated with enhanced functional connectivity of dorsolateral prefrontal cortex**

***Supplementary Material***

**Supplementary Methods**

*Sample size calculation.* The sample size was calculated to demonstrate a treatment difference of 6.0 in the MCCB composite score between the antipsychotics plus metformin group and antipsychotics alone group and a standard deviation of 7, according to the results from Mendella et al. ^1^ and clinical judgment. A minimum sample of 64 patients (42 in the antipsychotics plus metformin group and 22 in the antipsychotics alone group) would be required based on the following parameters: 80% power, a two-sided significance level of 5%, a 2:1 proportion, and 20% of patients lost to follow-up.

*Metabolic-related indexes.* The metabolic-related index includes weight; body mass index (BMI; kg/m^2^); fasting glucose; insulin; insulin resistance index (IRI), which was calculated based on the following formula of homeostasis assessment for insulin resistance model: fasting insulin (mU/L)×fasting glucose (mmol/L)/22.5; total cholesterol; triglyceride; high-density lipoprotein cholesterol (HDL-C); low-density lipoprotein cholesterol (LDL-C); liver and renal function, and blood routine tests were performed at each time point.

*MRI data acquisition and preprocessing.* We used a high-resolution T1-weighted 3D sequence and a gradient echo-planar imaging sequence to obtain brain structural and resting-state functional data, respectively. The participants were asked to avoid falling asleep, opening their eyes, or moving during the scanning procedure. The participants wore earmuffs designed to restrict head motion during scanning. The T1-weighted sequence parameters were as follows: repetition time (TR) = 8.25 ms, echo time (TE) = 3.78 ms, inversion time (TI) = 1100 ms, flip angle (FA) = 7°, matrix size = 256 × 256, field of view (FOV) = 256 mm × 256 mm, slice orientation = sagittal, slice number = 188, slice thickness = 1 mm, and gap = 0. The EPI sequence parameters were as follows: TR = 2000 ms, TE= 30 ms, FA = 90°, matrix size = 64 × 64, FOV = 220 mm × 220 mm, volume number = 240, slice orientation = transverse, slice number = 33, slice thickness = 4 mm, and gap = 0.6 mm.

Resting-state fMRI data were collected using a Philips 3T magnetic resonance scanner (Philips Healthcare, Best, The Netherlands) at Second Xiangya Hospital. The raw data were preprocessed using the Data Processing & Analysis of Brain Imaging toolbox (DPABI, V4.2, http://rfmri.org/dpabi) running on MATLAB software (The MathWorks, Inc., Natick, MA, USA)^2^. We discarded the first ten fMRI scanning volumes, then applied a slice-timing correction and head-motion realignment. The Montreal Neurological Institute standard template was used for image spatial normalization with a resampled 3 × 3 × 3 mm^3^ resolution. We then carried out a scrubbing procedure to remove volumes with a mean frame-wise displacement Power > 0.5 mm (37). In the antipsychotics plus metformin group, two patients were excluded after scrubbing because volumes remaining after scrubbing were required to be ≥ 70% of the original set. We used a 6 mm full-width at half-maximum Gaussian kernel to spatially smooth images. Then linear detrending was performed. Next, nuisance covariates were regressed out, including the white-matter signal, cerebrospinal-fluid signal, and the Friston-24 motion parameters. Then time series was temporally band-pass filtered with a 0.01-0.08 Hz range. The global signal was not removed as suggested in a previous study ^3^.

**Supplementary Results**

*Test of homogeneity of the variances.* Results of this section was presented in Supplementary Table 10 and Supplementary Table 11. Because the linear mixed model also applies to longitudinal data with heterogeneous variance, we did not compare the homogeneity of the variances of cognitive scores between the two groups.

*Safety evaluation.* Adverse events (grade ≥ 3) were more frequent in the antipsychotics plus metformin group than in the antipsychotics alone group. Two patients could not tolerate the maximum dose of metformin before the 12-week follow-up because of nausea and diarrhea, they were told to take 1,000 mg daily of metformin after consulting with our study team. Both patients reported well-tolerance to 1,000 mg daily of metformin. After 24 weeks, the most frequently observed adverse event was decreased appetite (15/45, 33.3% in the antipsychotics plus metformin group vs. 0% in the antipsychotics group, χ^2^ = 10.222, *P* = 0.001). No significant intergroup differences were observed in other adverse events. Abnormal liver function was more frequent in the antipsychotics alone group than in the antipsychotics plus metformin group (3/24, 12.5% vs. 2/45, 4.4%) (Supplementary Table 8).

**Supplementary Figures**


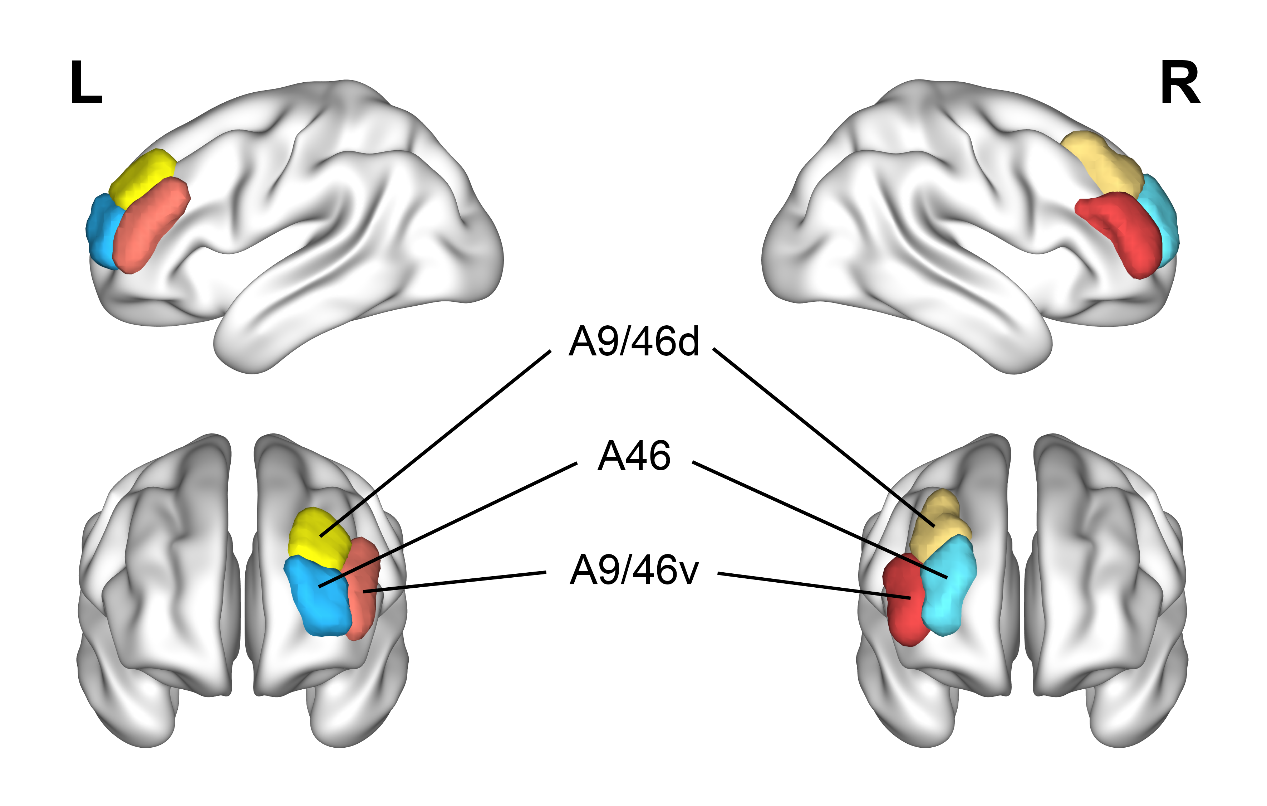


Supplementary Figure 1. General view of the six ROIs. As six seeds for seed-based voxel-wise functional connectivity. The A9/46d, A46 and A9/46v are the subregions of dorsolateral prefrontal cortex. Abbreviations: ROI, region of interest; L, left; R, right.

**Supplementary Tables**

| **Supplementary Table 1.** Demographic and clinical characteristics at baseline. | | | | |
| --- | --- | --- | --- | --- |
| **Characteristics** | **Antipsychotics plus metformin**  **(*N* = 45)**  **Mean (SD)** | **Antipsychotics alone**  **(*N* = 24)**  **Mean (SD)** | ***t* / χ^2^ / *Z*** | ***P*** |
| Sex, female/male | 38/7 | 18/6 | 0.400 | 0.527 ^a^ |
| Age, year | 22.89 (4.63) | 22.58 (5.28) | -0.672 | 0.502 |
| Education, year | 12.71 (3.00) | 12.54 (3.11) | -0.338 | 0.735 |
| Duration, month | 21.47 (15.01) | 28.71 (16.27) | -1.759 | 0.078 |
| CPZ-DDD, mg/day | 406.44 (237.73) | 380.21 (153.27) | -0.114 | 0.909 |
| Height, cm | 161.69 (7.51) | 162.83 (8.80) | -0.404 | 0.686 |
| Weight, kg | 67.00 (11.19) | 64.80 (9.39) | 0.821 | 0.414 |
| BMI, kg/m^2^ | 25.62 (3.79) | 24.43 (2.80) | -1.033 | 0.302 |
| Weight before medication, kg | 52.86 (9.88) | 52.13 (8.12) | -0.120 | 0.905 |
| Triglyceride, mmol/L | 1.51 (0.77) | 1.47 (0.74) | -0.025 | 0.980 |
| Total cholesterol, mmol/L | 4.50 (0.76) | 4.21 (0.61) | 1.584 | 0.118 |
| HDL-C, mmol/L | 1.28 (0.22) | 1.19 (0.27) | 1.543 | 0.128 |
| LDL-C, mmol/L | 2.70 (0.64) | 2.64 (0.49) | 0.393 | 0.696 |
| Fasting glucose, mmol/L | 4.99 (0.52) | 4.81 (0.57) | 1.302 | 0.198 |
| Insulin, mU/L | 17.56 (7.83) | 14.65 (6.81) | -1.445 | 0.148 |
| IRI | 4.02 (1.83) | 3.16 (1.73) | -1.892 | 0.059 |
| PANSS total score | 42.53 (5.40) | 42.38 (5.01) | -0.341 | 0.733 |
| CDSS | 0.27 (0.54) | 0.33 (0.64) | -0.325 | 0.745 |
| MCCB |  |  |  |  |
| Speed of processing | 37.93 (7.98) | 36.85 (7.32) | 0.554 | 0.582 |
| Attention/vigilance | 42.04 (8.37) | 41.62 (13.08) | 0.142 | 0.888 |
| Working memory | 40.51 (12.22) | 40.04 (9.79) | 0.162 | 0.872 |
| Verbal learning | 35.71 (9.71) | 36.54 (9.76) | -0.338 | 0.737 |
| Visual learning | 42.91 (11.89) | 40.54 (9.14) | 0.850 | 0.398 |
| Reasoning/problem solving | 40.29 (11.51) | 40.63 (13.55) | -0.109 | 0.914 |
| Social cognition | 41.00 (10.45) | 40.83 (9.21) | -0.429 | 0.668 |
| Composite score | 39.68 (5.78) | 38.97 (6.70) | 0.456 | 0.650 |
| ^a^ Fisher's precision probability test.  Abbreviations: CPZ, chlorpromazine; DDD, defined daily dose; BMI, body mass index; HDL-C, high-density lipoprotein cholesterol; LDL-C, low-density lipoprotein cholesterol; IRI, insulin resistance index; PANSS, Positive and Negative Syndrome Scale; CDSS, Calgary Depression Scale for Schizophrenia; MCCB, MATRICS Consensus Cognitive Battery; SD, standard deviation. | | | | |

| **Supplementary Table 2.** Treatment effects on MCCB cognitive domains of antipsychotics plus metformin group vs antipsychotics alone group at week 12/24 based on linear mixed-effects models. | | | | | |
| --- | --- | --- | --- | --- | --- |
| **MCCB** | **Week 12** | |  | **Week 24** | |
|  | **Metformin vs control Mean difference (95% CI)** | ***P*** |  | **Metformin vs control Mean difference (95% CI)** | ***P*** |
| Speed of processing | 4.69 (2.44 to 6.93) | < 0.001 |  | 3.75 (1.41 to 6.09) | 0.002 |
| Attention/vigilance | 0.93 (-2.78 to 4.64) | 0.616 |  | 1.38 (-2.13 to 4.89) | 0.435 |
| Working memory | 2.62 (-0.48 to 5.71) | 0.096 |  | 4.39 (0.72 to 8.06) | 0.020 |
| Verbal learning | 3.22 (-0.81 to 7.24) | 0.115 |  | 5.71 (2.06 to 9.36) | 0.003 |
| Visual learning | 3.15 (-0.89 to 7.18) | 0.124 |  | 7.08 (2.36 to 11.79) | 0.004 |
| Reasoning/problem solving | 3.86 (-0.53 to 8.25) | 0.083 |  | 3.68 (-1.16 to 8.52) | 0.133 |
| Social cognition | -2.19 (-7.17 to 2.79) | 0.382 |  | -1.50 (-6.47 to 3.47) | 0.548 |
| Composite score | 2.75 (1.29 to 4.20) | < 0.001 |  | 3.43 (1.83 to 5.02) | < 0.001 |
| Covariance structures: unstructured.  Metformin indicates antipsychotics plus metformin group, and control indicates antipsychotics alone group.  Abbreviations: MCCB, MATRICS Consensus Cognitive Battery; CI, confidence interval. | | | | | |

| **Supplementary Table 3.** Changes in MCCB cognitive scores at week 12 and week 24 for each group from linear mixed-effects models. | | | | | |
| --- | --- | --- | --- | --- | --- |
|  | **Antipsychotics plus metformin** | |  | **Antipsychotics alone** | |
| **MCCB** | **Estimated mean (SE)^a^** | **Mean difference (SE)^b^** |  | **Estimated mean (SE)^a^** | **Mean difference (SE)^b^** |
| Speed of processing |  |  |  |  |  |
| Baseline | 37.93 (1.19) | - |  | 36.85 (1.50) | - |
| Week 12 | 43.22 (1.06) | 5.29 (0.73)** |  | 38.09 (1.34) | 1.24 (1.01) |
| Week 24 | 45.82 (1.04) | 7.89 (0.73)** |  | 41.53 (1.50) | 4.69 (1.04)** |
| Attention/vigilance |  |  |  |  |  |
| Baseline | 42.04 (1.25) | - |  | 41.63 (2.67) | - |
| Week 12 | 46.39 (1.61) | 4.34 (1.21)** |  | 44.96 (2.73) | 3.33 (0.99)** |
| Week 24 | 49.58 (1.48) | 7.54 (1.06)** |  | 48.35 (2.32) | 6.73 (1.28)** |
| Working memory |  |  |  |  |  |
| Baseline | 40.51 (1.82) | - |  | 40.04 (2.00) | - |
| Week 12 | 43.38 (1.20) | 2.87 (1.07)** |  | 40.49 (1.83) | 0.45 (1.73) |
| Week 24 | 46.30 (1.49) | 5.79 (1.06)** |  | 41.57 (2.32) | 1.53 (2.03) |
| Verbal learning |  |  |  |  |  |
| Baseline | 35.71 (1.45) | - |  | 36.54 (1.99) | - |
| Week 12 | 39.86 (1.21) | 4.15 (1.28)** |  | 36.03 (2.25) | -0.51 (2.26) |
| Week 24 | 43.60 (1.32) | 7.89 (1.43)** |  | 37.66 (1.48) | 1.12 (1.82) |
| Visual learning |  |  |  |  |  |
| Baseline | 42.91 (1.77) | - |  | 40.54 (1.87) | - |
| Week 12 | 47.49 (1.27) | 4.58 (1.49)** |  | 42.72 (2.04) | 2.18 (1.69) |
| Week 24 | 50.01 (1.51) | 7.10 (1.89)** |  | 41.30 (1.95) | 0.76 (1.55) |
| Reasoning/problem solving |  |  |  |  |  |
| Baseline | 40.29 (1.72) | - |  | 40.63 (2.77) | - |
| Week 12 | 47.75 (1.81) | 7.46 (1.31)** |  | 44.92 (2.87) | 4.29 (1.75)* |
| Week 24 | 51.90 (1.61) | 11.62 (1.58)** |  | 49.40 (2.75) | 8.78 (1.95)** |
| Social cognition |  |  |  |  |  |
| Baseline | 41.00 (1.56) | - |  | 40.83 (1.88) | - |
| Week 12 | 39.44 (1.96) | -1.56 (1.47) |  | 41.02 (2.36) | 0.19 (1.83) |
| Week 24 | 40.33 (1.90) | -0.68 (1.43) |  | 41.16 (2.04) | 0.32 (1.82) |
| Composite score |  |  |  |  |  |
| Baseline | 39.68 (0.86) | - |  | 38.97 (1.37) | - |
| Week 12 | 43.75 (0.74) | 4.08 (0.50)** |  | 40.44 (1.21) | 1.47 (0.57)* |
| Week 24 | 46.56 (0.72) | 6.89 (0.56)** |  | 42.52 (1.29) | 3.55 (0.51)** |
| Covariance structures: unstructured.  ^a^ mean and SE at each time point were estimated by the simple effect of time from linear mixed-effects models.  ^b^ mean difference indicates the changes over 12 weeks and over 24 weeks estimated by the simple effect of time from linear mixed-effects models.  Abbreviations: MCCB, MATRICS Consensus Cognitive Battery; SE, standard error. * *P* < 0.05, ** *P* < 0.01. | | | | | |

| **Supplementary Table 4.** Treatment effects on MCCB cognitive domains of antipsychotics plus metformin group vs antipsychotics alone group at week 12/24 based on linear mixed-effects models after excluding participants with GDS less than 0.5. | | | | | |
| --- | --- | --- | --- | --- | --- |
| **MCCB** | **Week 12** | |  | **Week 24** | |
|  | **Metformin vs control Mean difference (95% CI)** | ***P*** |  | **Metformin vs control Mean difference (95% CI)** | ***P*** |
| Speed of processing | 4.66 (2.14 to 7.18) | < 0.001 |  | 4.60 (1.94 to 7.26) | 0.001 |
| Attention/vigilance | 1.02 (-3.46 to 5.50) | 0.647 |  | 0.10 (-4.10 to 4.31) | 0.960 |
| Working memory | 1.49 (-1.95 to 4.93) | 0.388 |  | 4.11 (0.21 to 8.02) | 0.040 |
| Verbal learning | 5.06 (1.05 to 9.07) | 0.015 |  | 6.94 (3.75 to 10.14) | < 0.001 |
| Visual learning | 3.50 (-1.27 to 8.26) | 0.146 |  | 7.95 (2.99 to 12.91) | 0.002 |
| Reasoning/problem solving | 2.36 (-2.82 to 7.54) | 0.363 |  | 5.44 (-0.23 to 11.11) | 0.060 |
| Social cognition | -1.30 (-7.15 to 4.55) | 0.656 |  | -0.05 (-5.45 to 5.35) | 0.985 |
| Composite score | 2.76 (1.05 to 4.47) | 0.002 |  | 4.05 (2.33 to 5.77) | < 0.001 |
| Covariance structures: unstructured.  Metformin indicates antipsychotics plus metformin group, and control indicates antipsychotics alone group.  Abbreviations: MCCB, MATRICS Consensus Cognitive Battery; GDS, global deficit score; CI, confidence interval. | | | | | |

| **Supplementary Table 5.** Changes in MCCB cognitive scores at week 12 and week 24 for each group from linear mixed-effects models after excluding participants with GDS less than 0.5. | | | | | |
| --- | --- | --- | --- | --- | --- |
|  | **Antipsychotics plus metformin** | |  | **Antipsychotics alone** | |
| **MCCB** | **Estimated mean (SE)** | **Mean difference (SE)^a^** |  | **Estimated mean (SE)** | **Mean difference (SE)^a^** |
| Speed of processing |  |  |  |  |  |
| Baseline | 35.99 (1.13) | - |  | 34.33 (1.31) | - |
| Week 12 | 41.56 (1.02) | 5.56 (0.84)** |  | 36.06 (1.25) | 1.73 (1.06) |
| Week 24 | 44.65 (1.14) | 8.66 (0.80)** |  | 39.09 (1.26) | 4.76 (1.21)** |
| Attention/vigilance |  |  |  |  |  |
| Baseline | 41.08 (1.18) | - |  | 37.63 (2.39) | - |
| Week 12 | 45.73 (1.69) | 4.65 (1.35)** |  | 41.36 (2.65) | 3.73 (1.23)** |
| Week 24 | 48.52 (1.51) | 7.44 (1.24)** |  | 45.38 (2.49) | 7.75 (1.24)** |
| Working memory |  |  |  |  |  |
| Baseline | 38.74 (1.85) | - |  | 38.74 (2.23) | - |
| Week 12 | 42.42 (1.28) | 3.68 (1.14)** |  | 40.76 (1.94) | 2.02 (1.96) |
| Week 24 | 44.60 (1.43) | 5.86 (1.02)** |  | 40.40 (2.53) | 1.67 (2.44) |
| Verbal learning |  |  |  |  |  |
| Baseline | 34.74 (1.61) | - |  | 34.42 (2.24) | - |
| Week 12 | 39.20 (1.31) | 4.47 (1.30)** |  | 33.31 (2.35) | -1.11 (2.61) |
| Week 24 | 42.96 (1.26) | 8.23 (1.26)** |  | 35.75 (1.48) | 1.33 (2.26) |
| Visual learning |  |  |  |  |  |
| Baseline | 41.53 (1.90) | - |  | 38.63 (1.97) | - |
| Week 12 | 46.28 (1.43) | 4.76 (1.65)** |  | 41.09 (2.27) | 2.46 (2.09) |
| Week 24 | 49.60 (1.56) | 8.08 (1.85)** |  | 40.10 (2.18) | 1.47 (1.81) |
| Reasoning/problem solving |  |  |  |  |  |
| Baseline | 38.68 (1.77) | - |  | 37.26 (3.02) | - |
| Week 12 | 46.03 (1.94) | 7.35 (1.47)** |  | 43.02 (3.42) | 5.76 (2.02)* |
| Week 24 | 51.69 (1.78) | 13.01 (1.75)** |  | 45.99 (2.92) | 8.73 (2.29)** |
| Social cognition |  |  |  |  |  |
| Baseline | 40.53 (1.81) | - |  | 39.00 (1.97) | - |
| Week 12 | 39.49 (2.17) | -1.03 (1.61) |  | 39.17 (2.80) | 0.17 (2.19) |
| Week 24 | 41.36 (2.11) | 0.83 (1.54) |  | 39.67 (2.34) | 0.67 (1.99) |
| Composite score |  |  |  |  |  |
| Baseline | 38.25 (0.81) | - |  | 36.52 (1.12) | - |
| Week 12 | 42.64 (0.73) | 4.39 (0.55)** |  | 38.48 (1.09) | 1.96 (0.56)** |
| Week 24 | 45.83 (0.74) | 7.58 (0.54)** |  | 40.36 (1.08) | 3.84 (0.62)** |
| Covariance structures: unstructured.  ^a^ mean and SE at each time point were estimated by the simple effect of time from linear mixed-effects models.  ^b^ mean difference indicates the changes over 12 weeks and over 24 weeks estimated by the simple effect of time from linear mixed-effects models.  Abbreviations: MCCB, MATRICS Consensus Cognitive Battery; GDS, global deficit score; SE, standard error. **P* < 0.05, ***P* < 0.01. | | | | | |

| **Supplementary Table 6.** Significant clusters showing time × group interaction effect in FC with DLPFC subregion as seeds between antipsychotics plus metformin group and antipsychotics alone group over 12 weeks. | | | | | | | |
| --- | --- | --- | --- | --- | --- | --- | --- |
| Seed | Cluster | Brain regions^a^ | Cluster size (voxel) | Peak coordinate (mm)^b^ | | | Peak *F* value^c^ |
|  |  |  |  | x | y | z |  |
| Left A9/46d | 1 | Right ACC | 71 | 9 | 42 | 24 | 25.124 |
|  |  | Right MCC |  |  |  |  |  |
| Left A46 | 1 | Right ACC | 62 | 9 | 36 | 21 | 24.501 |
| Right A46 | 1 | Right SFG | 75 (34 in right A9/46d)^d^ | 21 | 39 | 30 | 31.990 |
|  |  | Right MFG |  |  |  |  |  |
|  | 2 | MCC | 55 | -6 | -27 | 36 | 27.972 |
| ^a^ based on the automated anatomical atlas.  ^b^ peak coordinate refers to the peak voxel location of the significant cluster in the Montreal Neurological Institute space.  ^c^ *F* values of time × group interaction effect in two-way ANOVA.  ^d^ according to the Brainnetome atlas.  Abbreviations: FC, functional connectivity; DLPFC, dorsolateral prefrontal cortex; ACC, anterior cingulate cortex; MCC, middle cingulate cortex; SFG, superior frontal gyrus; MFG, middle frontal gyrus. | | | | | | | |

| **Supplementary Table 7.** The z-scored FC values of time × group interaction effects between DLPFC subregions and significant brain areas over 12 weeks. | | | | | | | | | | | | |
| --- | --- | --- | --- | --- | --- | --- | --- | --- | --- | --- | --- | --- |
|  | **Antipsychotics plus metformin (*N* = 25)** | | | | |  | **Antipsychotics alone (*N* = 17)** | | | | |  |
| **FC** | **Baseline mean (SD)** | **Week 12 mean (SD)** | **Paired *t*** | **Paired *P*** | **Δ_12-0_ mean (SD)**^a^ |  | **Baseline mean (SD)** | **Week 12 mean (SD)** | **Paired *t*** | **Paired *P*** | **Δ_12-0_ mean (SD)^a^** | **Δ_12-0_ *P***^a^ |
| A9/46d.L-ACC.R/MCC.R | 0.16(0.12) | 0.29(0.17) | 4.085 | < 0.001 | 0.13(0.16) |  | 0.3(0.19) | 0.16(0.17) | -3.598 | 0.002 | -0.13(0.15) | < 0.001 |
| A46.L-ACC.R | 0.27(0.18) | 0.41(0.09) | 3.929 | < 0.001 | 0.14(0.18) |  | 0.42(0.15) | 0.28(0.17) | -3.985 | 0.001 | -0.14(0.15) | < 0.001 |
| A46.R-SFG.R/MFG.R | 0.15(0.2) | 0.35(0.12) | 5.746 | < 0.001 | 0.2(0.17) |  | 0.29(0.22) | 0.14(0.29) | -4.652 | < 0.001 | -0.15(0.14) | < 0.001 |
| A46.R-MCC | 0.28(0.13) | 0.43(0.19) | 3.808 | < 0.001 | 0.14(0.19) |  | 0.42(0.12) | 0.27(0.15) | -3.310 | 0.004 | -0.15(0.18) | < 0.001 |
| ^a^ indicates changes from week 12 to baseline.  Abbreviations: FC, functional connectivity; DLPFC, dorsolateral prefrontal cortex; ACC, Anterior cingulate cortex; MCC, middle cingulate cortex; SFG, superior frontal gyrus; MFG, middle frontal gyrus; SD, standard deviation; L/R, left/right. | | | | | | | | | | | | |

| **Supplementary Table 8.** Correlations between changes of FC with DLPFC subregions as seeds and changes of MCCB cognitive domains over 12 weeks. | | | | | | | | | |
| --- | --- | --- | --- | --- | --- | --- | --- | --- | --- |
| **FC** |  | Speed of processing | Attention/  vigilance | Working memory | Verbal learning | Visual learning | Reasoning/problem solving | Social cognition | Composite score |
| A9/46d.L-ACC.R/MCC.R | *r* | 0.273 | 0.181 | 0.325 | 0.019 | 0.102 | 0.217 | -0.215 | 0.330 |
|  | ***P*** | 0.080 | 0.251 | 0.036 | 0.905 | 0.521 | 0.168 | 0.172 | 0.033 |
| A46.L-ACC.R | *r* | 0.221 | 0.130 | 0.290 | 0.203 | 0.218 | 0.239 | -0.198 | 0.395 |
|  | ***P*** | 0.159 | 0.413 | 0.063 | 0.198 | 0.165 | 0.127 | 0.209 | 0.010 |
| A46.R-SFG.R/MFG.R | *r* | 0.241 | 0.061 | -0.093 | 0.173 | 0.024 | 0.373 | 0.028 | 0.327 |
|  | ***P*** | 0.125 | 0.699 | 0.557 | 0.273 | 0.879 | 0.015 | 0.862 | 0.034 |
| A46.R-MCC | *r* | 0.298 | 0.138 | 0.069 | 0.114 | 0.229 | 0.191 | -0.040 | 0.392 |
|  | ***P*** | 0.055 | 0.384 | 0.665 | 0.473 | 0.144 | 0.227 | 0.804 | 0.010 |
| Abbreviations: MCCB, MATRICS Consensus Cognitive Battery; FC, functional connectivity; DLPFC, dorsolateral prefrontal cortex; ACC, Anterior cingulate cortex; MCC, middle cingulate cortex; SFG, superior frontal gyrus; MFG, middle frontal gyrus; SD, standard deviation; L/R, left/right.. | | | | | | | | | |

| **Supplementary Table 9.** Adverse events between two groups at week 24. | | | | | | | | |
| --- | --- | --- | --- | --- | --- | --- | --- | --- |
|  | **Antipsychotics plus metformin (*N* = 45)** | |  | **Antipsychotics alone (*N* = 24)** | | **χ^2^** | ***P*** |  |
|  | ***N*** | **%** |  | ***N*** | **%** |  |  |  |
| Somnolence | 3 | 6.7% |  | 2 | 8.3% | 0.000 | 1.000 |  |
| Abnormal hemogram | 2 | 4.4% |  | 0 | 0% |  | 0.540^a^ |  |
| Abnormal liver function | 2 | 4.4% |  | 3 | 12.5% | 0.550 | 0.458 |  |
| Nausea and vomit | 2 | 4.4% |  | 0 | 0% |  | 0.540^a^ |  |
| Diarrhea | 1 | 2.2% |  | 0 | 0% |  | 1.000^a^ |  |
| Tachycardia | 3 | 6.7% |  | 1 | 4.2% | 0.000 | 1.000 |  |
| Decreased appetite | 15 | 33.3% |  | 0 | 0% | 10.222 | 0.001 |  |
| ^a^ Fisher's precision probability test. | | | | | | | |  |

| **Supplementary Table 10.** Test of homogeneity of baseline measurement variances. | | |
| --- | --- | --- |
| **Characteristics** | **Levene's statistic (Based on mean)** | ***P*** |
| Age, year | 1.062 | 0.306 |
| Education, year | 0.196 | 0.660 |
| Duration, month | 0.958 | 0.331 |
| CPZ-DDD, mg/day | 2.651 | 0.108 |
| Height, cm | 0.098 | 0.756 |
| Weight, kg | 0.604 | 0.440 |
| BMI, kg/m^2^ | 2.609 | 0.111 |
| Weight before medication, kg | 0.961 | 0.330 |
| Triglyceride, mmol/L | 0.016 | 0.900 |
| Total cholesterol, mmol/L | 1.394 | 0.242 |
| HDL-C, mmol/L | 1.245 | 0.269 |
| LDL-C, mmol/L | 2.580 | 0.113 |
| Fasting glucose, mmol/L | 1.300 | 0.259 |
| Insulin, mU/L | 0.748 | 0.390 |
| IRI | 0.393 | 0.533 |
| PANSS total score | 0.102 | 0.750 |
| CDSS | 0.907 | 0.344 |
| MCCB |  |  |
| Speed of processing | 0.195 | 0.661 |
| Attention/vigilance | 6.855 | 0.011 |
| Working memory | 0.457 | 0.501 |
| Verbal learning | 0.115 | 0.735 |
| Visual learning | 2.119 | 0.150 |
| Reasoning/problem solving | 1.554 | 0.217 |
| Social cognition | 0.965 | 0.330 |
| Composite score | 0.613 | 0.436 |
| Abbreviations: CPZ, chlorpromazine; DDD, defined daily dose; BMI, body mass index; HDL-C, high-density lipoprotein cholesterol; LDL-C, low-density lipoprotein cholesterol; IRI, insulin resistance index; PANSS, Positive and Negative Syndrome Scale; CDSS, Calgary Depression Scale for Schizophrenia; MCCB, MATRICS Consensus Cognitive Battery. | | |

| **Supplementary Table 11.** Test of homogeneity of metabolic measurement variances. | | |
| --- | --- | --- |
| **Characteristics** | **Levene's statistic (Based on mean)** | ***P*** |
| Changes between baseline and week 12 |  |  |
| Weight, kg | 1.192 | 0.279 |
| BMI, kg/m^2^ | 1.294 | 0.260 |
| Triglyceride, mmol/L | 2.407 | 0.126 |
| Total cholesterol, mmol/L | 0.084 | 0.774 |
| HDL-C, mmol/L | 0.672 | 0.416 |
| LDL-C, mmol/L | 1.073 | 0.304 |
| Fasting glucose, mmol/L | 3.082 | 0.084 |
| Insulin, mU/L | 6.005 | 0.017 |
| IRI | 4.140 | 0.046 |
| Changes between baseline and week 24 |  |  |
| Weight, kg | 0.184 | 0.669 |
| BMI, kg/m^2^ | 0.239 | 0.626 |
| Triglyceride, mmol/L | 3.265 | 0.076 |
| Total cholesterol, mmol/L | 0.179 | 0.674 |
| HDL-C, mmol/L | 1.206 | 0.276 |
| LDL-C, mmol/L | 0.259 | 0.613 |
| Fasting glucose, mmol/L | 0.230 | 0.634 |
| Insulin, mU/L | 6.460 | 0.014 |
| IRI | 4.100 | 0.047 |
| Abbreviations: BMI, body mass index; HDL-C, high-density lipoprotein cholesterol; LDL-C, low-density lipoprotein cholesterol; IRI, insulin resistance index. | | |

**References**

**1.** Mendella PD, Burton CZ, Tasca GA, Roy P, Louis LS, Twamley EW. Compensatory cognitive training for people with first-episode schizophrenia: results from a pilot randomized controlled trial. *Schizophrenia research* 2015;162(1-3):108-111.

**2.** Yan CG, Wang XD, Zuo XN, Zang YF. DPABI: Data Processing & Analysis for (Resting-State) Brain Imaging. *Neuroinformatics* Jul 2016;14(3):339-351.

**3.** Hahamy A, Calhoun V, Pearlson G, Harel M, Stern N, Attar F, Malach R, Salomon R. Save the global: global signal connectivity as a tool for studying clinical populations with functional magnetic resonance imaging. *Brain connectivity* 2014;4(6):395-403.
